# Patient-reported outcomes in a Chinese cohort of osteogenesis imperfecta unveil psycho-physical stratifications associated with clinical manifestations

**DOI:** 10.1186/s13023-022-02394-7

**Published:** 2022-06-28

**Authors:** Peikai Chen, Zhijia Tan, Anmei Qiu, Shijie Yin, Yapeng Zhou, Zhongxin Dong, Yan Qiu, Jichun Xu, Kangsen Li, Lina Dong, Hiu Tung Shek, Jingwen Liu, Eric H. K. Yeung, Bo Gao, Kenneth Man Chee Cheung, Michael Kai-Tsun To

**Affiliations:** 1 Department of Orthopedics and Traumatology, The University of Hong Kong-Shenzhen Hospital (HKU-SZH), Shenzhen, 518053 Guangdong China; 2 Department of Physiotherapy, The University of Hong Kong-Shenzhen Hospital (HKU-SZH), Shenzhen, 518053 Guangdong China; 3 Department of Orthopedics and Traumatology, The University of Hong Kong, Pok Fu Lam, Hong Kong; 4 School of Biomedical Sciences, The University of Hong Kong, Pok Fu Lam, Hong Kong

**Keywords:** Osteogenesis imperfecta, Rare disease, Psycho-physical, PROM, Genetic testing, Patient stratification, Cross-sectional, Longitudinal, Children, Adults

## Abstract

**Background:**

Osteogenesis imperfecta (OI) is a rare congenital disorder of the skeletal system, inflicting debilitating physical and psychological distress on patients and caregivers. Over the decades, much effort has been channeled towards understanding molecular mechanisms and developing new treatments. It has recently become more apparent that patient-reported outcome measurements (PROM) during treatment, healing and rehabilitation are helpful in facilitating smoother communication, refining intervention strategies and achieving higher quality of life. To date, systematic analyses of PROM in OI patients remain scarce.

**Results:**

Here, utilizing a PROM Information System, we report a cross-sectional and longitudinal study in a southern Chinese cohort of 90 OI patients, covering both the child and adult age-groups. In the child group where both self and parental surveys were obtained, we identified two clusters of comparable sizes showing different outlooks in physical mobility and emotional experiences. One cluster (Cluster 1) is more negative about themselves than the other (Cluster 2). A concordance of 84.7% between self and parental assessments was recorded, suggesting the stability and validity of PROM-based stratification. Clinical subtyping, deformity, leg length discrepancy, and limited joint mobility were significantly associated with this stratification, with Cluster 1 showing higher percentages of severe phenotypes than Cluster 2. Since OI is a genetic disorder, we performed genetic testing on 72 of the 90 patients, but found no obvious association between genotypes and the PROM stratification. Analyses of longitudinal data suggested that patients tended to stay in the same psychological state, in both clusters. Adult patients also showed a continuous spectrum of self-evaluation that matches their clinical manifestations.

**Conclusion:**

By systematically analyzing patient-reported outcomes, our study demonstrated the link between the sociopsychological wellbeing of OI patients, and their clinical manifestations, which may serve as the basis for evaluating clinical interventions and help achieve better patient-centric medical practices. The lack of genotype-PROM association may be due to the diverse mutational spectrum in OI, which warrants further investigation when a larger sample size is available.

**Supplementary Information:**

The online version contains supplementary material available at 10.1186/s13023-022-02394-7.

## Introduction

Osteogenesis imperfecta (OI) is a heterogeneous group of inherited skeletal dysplasia and connective tissue disorders with a prevalence of 0.3–1.5 per 10,000 live births [1, 2]. Individuals with OI are characterized by low bone mass and high bone fragility, resulting in susceptibility to long bone deformity, fracture and vertebral compression [3]. A wide spectrum of secondary features, including blue sclerae, dentinogenesis imperfecta, scoliosis, hearing loss, muscle weakness, ligamentous joint laxity and basilar invagination, were present in certain subsets of patients [1, 4]. Most OI patients are heterozygous for dominant mutations in *COL1A1* or *COL1A2*, which encode the main components of extracellular matrix in bones and skin [5, 6]. To date, genetic analyses have identified over 17 other OI-associated genes, which mainly play roles in the post-translational modification of collagens, bone mineralization or osteoblast differentiation [1, 7–10].

The multitude of affected genes and the unlimited possibilities of mutation patterns match the broad spectrum of clinical severities observed in OI, which range from occasional fractures, to dwarfism, and perinatal lethality. Based on clinical and hereditary features, Sillence et al*.* classified OI into four major types: type I (mild with bone fragility and blue sclerae), type II (perinatal lethal), type III (progressive deformity) and type IV (short stature, bone deformity and dentinogenesis imperfecta) [10–12]. Until lately, much focus has been placed on establishing the genotype–phenotype association [2, 13, 14], in the hope that the mechanistic aspect of the disease can better inform diagnoses and improve treatment outcomes. As such, clinical interventions and scientific research have mainly focused on the orthopedic [15] and pharmacological outcomes of OI patients. Nonetheless, the phenotypes and clinical outcomes are still objective observations, which do not reflect the subjective wellbeing of OI patients suffering from this lifelong debilitating condition. The diverse range of clinical issues inflicts major physical and psychological distress on OI patients [16–19], greatly compromising their life quality and causing heavy socioeconomic burdens [20, 21].

Quality of life (QoL) is a World Health Organization certified concept that measures the overall physical and mental wellbeing of an individual [16, 20]. Several studies have assessed health related QoL in OI patients and caregivers using generic instruments, including Short Form Health Survey (SF-36) [16, 17], EQ-5D-5 [18] and WHOQOL questionnaires [19]. Such efforts improved the communications between clinicians and patients and the ensuing decision-making, and increased patient satisfaction overall.

The various standards used to assess QoL cause difficulty in cross-study comparisons and interpretation, and many of the studies were focused on adults only [16–18, 22]. Almost all of these studies were cross-sectional [22]. Longitudinal data, which are highly valuable for such chronic conditions, remain scarce. SF-36, which only has 36 question items, is the most frequently used questionnaire. In the past decade, PROMIS (Patient-Reported Outcomes Measurement Information System) gained traction for its extensive question bank totaling over 20,000 survey items and covering a wide range of mental and physical issues [23]. Carrying on from their earlier initiative [24], a pilot study leveraging the power of PROMIS [25] was conducted on 290 OI patients, 198 of whom were adults. The study showed that the data averted the least desirable characteristics of floor and ceiling effects that commonly infest survey studies, hence demonstrating the feasibility and potential of PROMIS in OI. Nonetheless, in addition to a number of issues raised by the authors themselves, including gender disparity and insufficient data for non-white ethnicities, more in-depth analyses remain to be seen. For example, patient stratification in terms of PROMIS, and the connections between such stratification and the clinical and genetic features would help gain more insights into the principal factors impacting the patients’ QoL [26]. Particularly, OI is a genetic disorder, and genetic information is a key factor in OI diagnosis. How the genotypes may impact QoL and inform clinical decisions remains elusive. It is of interest to ascertain if certain genetic disposition predicts poorer or better prognosis. Assessments by parents and children and their concordance, and longitudinal follow-ups are also lacking. Some of the question items are similar, thus proper de-correlation is needed to bring out the principal patterns underlying patient stratifications.

In this study, we presented a PROMIS dataset of children and adults collected from a Southern Chinese OI cohort recruited by our hospital (HKU-SZH), a tertiary general hospital in China. Systematic analyses based on advanced statistical and machine learning approaches were conducted to unravel the patterns, structures and distributions of various self-assessment domains, identifying groups of patients with similar psycho-physical states. We demonstrated the stability of such stratifications from self and parental assessments, and from longitudinal data. We then explored the connections between the identified PROMIS patient-groups, and the objective genetic and clinical features, to identify the most relevant objective factors explaining the subjective feelings. These patient-centric data, methodological developments and novel findings will enrich the toolboxes for OI research, deepen our understanding of the disease, and benefit the life quality of the OI community as we move towards a more patient-centric diagnostic and therapeutic direction.

## Methods

### Subject recruitment and data collection

Ninety OI patients were recruited from our hospital (HKU-SZH) to participate in the current study since July of 2020 with IRB approval and informed consents. A web-based platform was developed based on the RedCap system [27], by which both clinical data and subjective evaluation data were recorded. The child group (aged below 18) were invited to fill in a form with 57 items (20 items related to physical capabilities, including mobility and pain; and 37 items related to sociopsychological status, including anxiety, depression, and peer relations) (Additional file 1). Their parents or proxies were invited to fill in a form with 49 items, including 16 items on physical capabilities and 33 items on sociopsychological states (Additional file 1). The form was adapted from the item banks of PROMIS (Item Bank v.1.0 and Pediatric Item Bank v2.0) [23, 28] and translated into Chinese language. A social worker (Y.Q.) was involved in explaining the questions to preschool children. Most of the items (except overall pain score, ranging from 0 to 10) were scored from 1 to 5, with 5 being the most positive for mobility and peer relations, and most negative for the other items. The surveys were conducted on the first day of inpatient admission to the hospital before surgical and/or drug treatments.

### Clinical features and muscle strengths

Detailed clinical features, including X-ray images, genetic reports, fracture history, heights, bone mineralization density (BMD), sclera and teeth issues, were documented by a panel of clinicians (M.K.T.T., S.J.Y., Y.P.Z., Z.X.D., J.C.X., K.S.L.). The BMD were measured by the Discovery DXA system (Hologic Inc., Massachusetts) at HKU-SZH. The BMDs at the lumbar region of the spine were used. The muscle strengths were rated by a registered physiotherapist (E.H.K.Y.), based on a representative lower limb strength for each patient. The scale is as below: 5: almost normal strength, 4: good strength against resistance; 3: reasonable strength against gravity; 2: poor strength, cannot against gravity; 1: almost no strength.

### Targeted amplicon sequencing

Peripheral blood samples of the patients were taken and processed before being sent for targeted amplicon sequencing of a panel of 24 genes associated with OI (DynastyGene, Shanghai). The 24 genes were: *COL1A1*, *COL1A2*, *IFITM5*, *SERPINF1*, *CRTAP*, *P3H1*, *PPIB*, *SERPINH1*, *FKBP10*, *PLOD2*, *BMP1*, *SP7*, *TMEM38B*, *WNT1*, *CREB3L1*, *SPARC*, *FAM46A*, *MBTPS2*, *MESD*, *CCDC134*, *P4HB*, *SEC24D*, *PLS3*, and *LRP5*. The samples were sequenced on the NovaSeq 6000 platform (Illumina Inc.). The raw data (150 bp paired-end) were aligned to the human reference sequence GRCh37/hg19 by the BWA aligner (version 0.7.17-r1188) [29] with default parameters. The GATK toolkit (version 4.0.4.0) [30] was then used to call the variants from the aligned BAM files. The results were annotated by SNPeff [31] and ANNOVAR [32], and deposited in VCF (variant calling format) files to be reviewed by our team of clinicians and geneticists.

### Data analyses

The data was in integer format ranging from 1 to 5 (for most items except pain scores ranging from 0 to 10). Although the empirical marginal density estimation on the patients or PROMIS items, or their aggregated distributions (Figs. 3F, 4C, 7B) did display certain degrees of skewness, given the narrow dynamic ranges, a Gaussian approximation was considered appropriate. As such the data were directly subjected to principal component analysis (PCA), which relies on a Gaussian or quasi-Gaussian assumption. Let ${x}_{i}^{t}\in {\mathbb{Z}}^{m}$ be the PROMIS outcome of patient $i (=\mathrm{1,2},\dots ,n)$ at time $t (=\mathrm{1,2},\mathrm{3,4})$, where $m$ is the number of answers per survey, we performed singular value decomposition on the matrix $${{\varvec{X}}}^{1}={[\begin{array}{ccc}{x}_{1}^{1}& \cdots & {x}_{n}^{1}\end{array}]}^{\boldsymbol{\top }}\in {\mathbb{R}}^{n\times m}$$, which represents the outcomes from the first PROMIS and has been column-wise zero-meaned (but not unit-varianced to avoid exaggerating less dispersed items), to obtain ${{\varvec{X}}}^{1}={\varvec{U}}\boldsymbol{\Sigma }{{\varvec{V}}}^{\boldsymbol{\top }}$ where $${{\varvec{V}}}^{\boldsymbol{\top }}={[\begin{array}{ccc}{v}_{1}& \cdots & {v}_{m}\end{array}]}^{\boldsymbol{\top }}\in {\mathbb{R}}^{m\times m}$$, $\boldsymbol{\Sigma }=diag({\delta }_{1},{\delta }_{2},\dots ,{\delta }_{m})$ and ${\delta }_{1}\ge {\delta }_{2}\ge \dots \ge {\delta }_{m}$. Then, ${v}_{1}\in {\mathbb{R}}^{m\times 1}$ is the first principal component (PC), ${v}_{2}$ the second, and so on. The percentage of variance explained for PC $j$ is given by ${({\delta }_{j})}^{2}/{\sum }_{k}{({\delta }_{k})}^{2}$. The new projections of the first PROMIS onto PC1 and PC2 are given by: ${{\varvec{Y}}}^{1}={{\varvec{X}}}^{1}[{v}_{1};\boldsymbol{ }{v}_{2}]$. And the projections of longitudinal PROMIS on the first PROMIS are given by ${{\varvec{Y}}}^{2}={{\varvec{X}}}^{2}[{v}_{1};\boldsymbol{ }{v}_{2}]$, ${{\varvec{Y}}}^{3}={{\varvec{X}}}^{3}[{v}_{1};\boldsymbol{ }{v}_{2}]$ and so on.

In correcting heights and BMD for ages, we first performed a linear regression of: $y=\alpha +\beta x+\varepsilon$, where $y$ are heights or BMD, $x$ are the ages (in years), $\alpha$ the intercepts, $\beta$ the slope, and $\varepsilon$ the error term with normal distribution. The coefficients $\widehat{\alpha }$ and $\widehat{\beta }$ were fitted by maximum likelihood estimation (equivalent to least square under current assumption). We then used the fitted residuals (called ‘partial residuals’) [33]: $\widehat{\varepsilon }=y-\widehat{y}$, where $\widehat{y}$ are the fitted values given by $\widehat{y}=\widehat{\alpha }+\widehat{\beta }x$, to compare against other variables (e.g., gender or patient clusters) by Analysis of Variance (ANOVA) [34].

For one- and two-dimensional density estimations, we used the kernel estimation method with default parameter. Heatmap clustering was performed with default parameters using the d3heatmap package of R. Associations between two categorical variables (e.g., patient clusters vs. genotypes) were tested by the Pearson’s ${\chi }^{2}$ test. All statistics were conducted on the R platform, version 4.0.3.

## Results

### Demographical characteristics

**Fig. 1:**
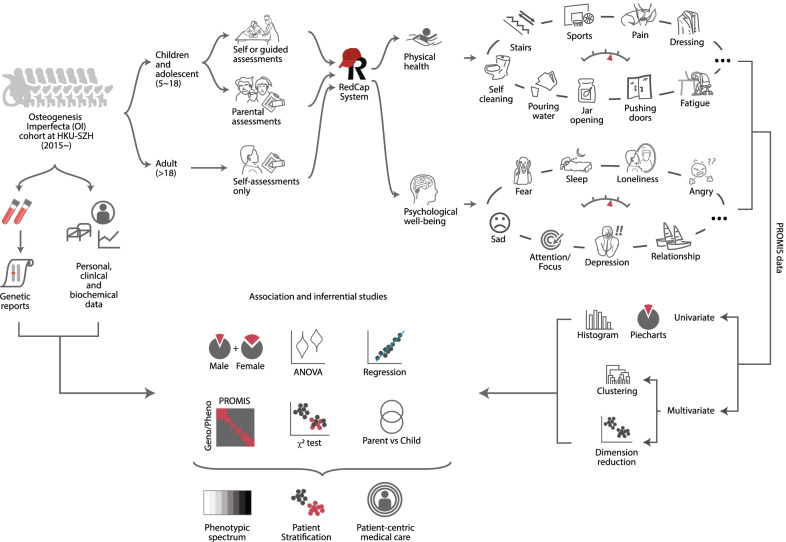
Schematic diagram showing the study design and data flow

A total of 90 OI patients were recruited consecutively and on a first-come, first-served basis in the current study since July 2020 (Methods). Both objective (personal, genetics, and clinical) and subjective data (PROMIS survey) [23] were collected for analyses, as depicted in a diagram (Fig. 1).

**Fig. 2:**
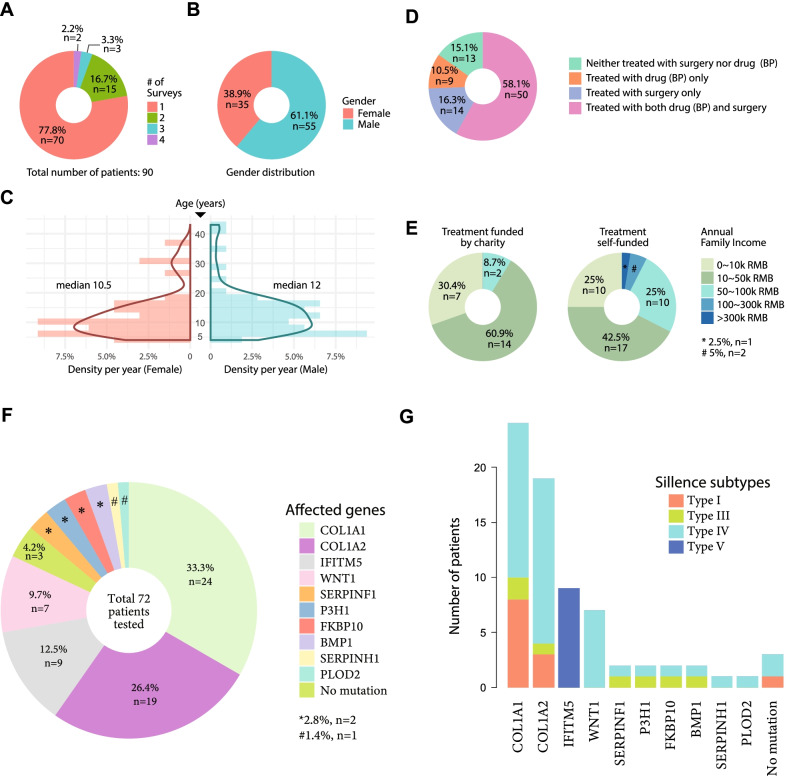
Overview of the current patient cohort. **A** Pie chart showing the frequency of PROMIS tables provided by the 90 patients. **B** Pie chart showing the gender distribution. **C** Pyramid histograms showing the age distribution in the two genders. Solid curves represent fitted kernel density estimations. **D** Treatment strategies among the 86 patients with records. BP: bisphosphonate. **E** Self-reported socioeconomic situations of the patients’ families, stratified by the sources of medical expenses, among the 63 patients where such data were available. RMB is the Chinese currency. **F** Pie chart showing the distributions of affected genes among the 59 patients that underwent genetic screening on a panel of 24 genes for targeted sequencing. **G** Bar chart showing the distribution of Sillence subtypes among the different genotypes

The majority of patients in our cohort were recruited from southern China except for 4 patients from northern China (Additional file 2). Seventy patients (77.8%) completed the questionnaire once, while 20 (22.2%) were involved in longitudinal surveys (Fig. 2A). Among the patients with longitudinal surveys, the median intervals between the first and second, the second and third, and the third and fourth surveys were 84, 59, and 36 days, respectively (Additional file 2). Similar intervals were recorded for the parental surveys (70, 61 and 41.5 days, respectively) (Additional file 2). Gender-wise, 35 (38.9%) patients were female and 55 (61.1%) were male (Fig. 2B). The ages of the patients ranged from 4 to 43 years (median 12 years). The median ages for females and males were 10.5 years and 12 years, respectively (Fig. 2C). We categorized the patients into the child group (below 18 years; n = 74) and adult group (18 years or above; n = 16) (Table 1).

**Table 1:** Demographical characteristics

	Child group (< 18 years)	Adult group (≥ 18 years)	*P* value*
Numbers of patients	74	16	
Age (years)	10.7 (IQR 7.5 ~ 14)	26.7 (IQR 19 ~ 31)	
Male %	60.8% (45 out of 74)	62.5% (10 out of 16)	1.0
Sillence subtypes			0.015
I	n = 7^a^ (11.1%) ^b^	n = 1 (6.7%)	
III	n = 8 (12.7%)	n = 7 (46.7%)	
IV	n = 40 (63.5%)	n = 6 (40%)	
V	n = 8 (12.7%)	n = 1 (6.7%)	
Number of fractures per year	1.1 (IQR 0.6 ~ 1.4)	0.5 (IQR 0.2 ~ 0.5)	0.003
Leg length discrepancy (LLD)	61.8% (42 out of 68)	92.8% (13 out of 14)	0.052
Dentinogenesis imperfecta	58.4% (38 out of 65)	50% (7 out of 14)	0.78
Limited joint mobility	22.4% (15 out of 67)	42.8% (6 out of 14)	0.21
Hearing loss	5.9% (4 out of 68)	7.1% (1 out of 14)	1.0
Scoliosis	47.8% (32 out of 67)	73.3% (11 out of 15)	0.13
Radial head dislocation	18.2 (12 out of 66)	28.6% (4 out of 14)	0.61
Sclera	73.1% (49 out of 67)	71.4% (10 out of 14)	1.0
Muscle strengths^c^	4.0 (IQR 3.7 ~ 4.3)	3.6 (IQR 3.3 ~ 4.0)	0.19
2	n = 3^a^	n = 0	
2+	n = 1	n = 0	
3	n = 6	n = 1	
3+	n = 7	n = 1	
4−	n = 1	n = 0	
4	n = 19	n = 2	
4+	n = 4	n = 2	
5	n = 2	n = 1	

^*^Based on Pearson χ^2^ testing (for categorical variables) or Wilcoxon rank-sum testing (for continuous variables). *IQR* inter-quartile range

^a^n is the number of patients

^b^Out of patients of whom Sillence type information was available

^c^3+ is better than 3 but poorer than 4 and 4-

We categorized the patients by the Sillence classification [10–12] (Fig. 2G). Briefly, 8 (10.2%), 15 (19.2%), 46 (59.0%), and 9 (11.5%) patients were classified as subtypes I, III, IV and V, respectively (Table 1). We documented a range of clinical features as well (Methods) (Table 1). We observed a lower fracture frequency ($P=$ 0.003) in the adult group, which might be related to more treatments and more self-awareness in fracture preventions in this group. Albeit not statistically significant, more leg length discrepancy, scoliosis and joint issues were observed in the adult group, which might be related to disease progression (Table 1). Congenital conditions, such as hearing and tooth issues and sclera, did not display age disparity (Table 1). Muscle strengths for 50 patients were available. To facilitate comparisons, only one data-point representing the lower limb strength was recorded per patient (Methods). With an average of 3.7 (IQR 3.3 ~ 4.0), we found the patients overall have good muscle strength against resistance, with no difference ($P=$ 0.19) observed between the two age-groups (Table 1).

Most of the patients (~ 85%) had been treated with surgery (osteotomy) and/or drugs (mainly bisphosphonates) before their first PROMIS survey. Fifty patients (58.1%) received both drug injection and osteotomy at least once (Fig. 2D). The financial sources of treatment included charity (38.5%) and self-financing (61.5%), the latter of which corresponded to higher annual family incomes (Fig. 2E).

### Targeted sequencing revealed a mutation spectrum in OI-related genes

The peripheral blood samples of 72 patients were collected for genetic tests on a panel of OI-related genes (Methods). Genetic test results of 35 patients were recently published [35], while those of the remaining 37 patients were first reported in the current study (Additional file 3). We detected pathogenic variants in 11 genes, among which mutations in *COL1A1* (n = 24, 33.3%) and *COL1A2* (n = 19, 26.4%) accounted for a combined 59.7% (n = 43) (Additional file 3; Fig. 2F), which was lower than previous reports in western countries [2, 5] but higher than in a recent Indian study [36]. Of note, deleterious mutations in *IFITM5* and *WNT1* were the major non-*COL1A1/2* mutations, being detected in 12.5% (n = 9) and 9.7% (n = 7) of the cohort, respectively (Fig. 2F). Mutations were detected with low frequencies in the remaining genes, including 2 cases in *BMP1*, *FKBP10*, *P3H1*, and *SERPINF1* each, and 1 case each in *SERPINH1* and *PLOD2* (Fig. 2F). No pathogenic mutation was detected in 3 patients (Fig. 2F). In terms of inheritance patterns, 54 patients (72.2%) fell in the autosomal dominant category, and 17 (23.6%) in the autosomal recessive category.

### PROMIS by children and adolescents reflected two different psychophysical states

**Fig. 3:**
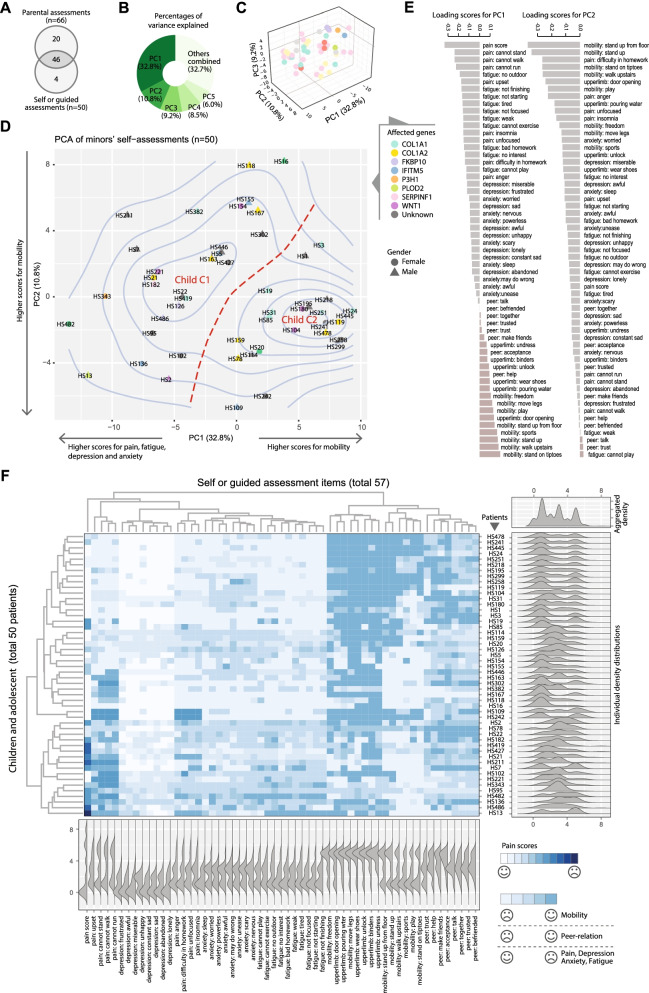
PCA and clustering analyses of child and adolescent PROMIS data. **A** Venn diagram showing the overlap of self and parental assessment cases. **B** Piechart showing the percentages of variance explained by principal component analysis (PCA). **C** A three-dimensional scatter plot showing the projections onto the top three components. Each dot represents one patient. **D** Scatter plot showing the first two PCs, with density contours. Red curves indicate saddle and valley between the two peaks/clusters. **E** Bar charts showing the ordered loading scores for the first and second PC. **F** Heatmap with clustering showing data in their original values. Marginal ridge plots show the marginal densities either per-patient (row-wise) or per PROMIS-item (column-wise), with an aggregated density for all data points placed on the top right corner. The meanings of the colors are explained by the smile or sad face symbols. The PROMIS items are abbreviated by their categories and a representative keyword. Refer to Additional file 1 for corresponding questions in full

A total of 74 children and adolescents (below 18 years) participated in the current PROMIS survey, 4 of whom did not complete surveys and were not analyzed. Among the 70 patients, 46 had both self and parental evaluation, 4 had self-assessment only, and 20 were assessed by their parents or proxies only (Fig. 3A). We noticed that some of the question items were similar to each other, which can be addressed by de-correlation methods such as principal component analysis (PCA). To extract the main patterns, we first performed a PCA on the 50 self-assessed outcomes (Fig. 3B–D). The top 5 principal components (PCs) explained over two thirds (67.2%) of the total variance in the data (Fig. 3B). The first PC was the most dominant, explaining almost one third of the variance. A three-dimensional chart showed that the top three PCs in combination distinguished the samples into two major clusters (Fig. 3C). A scatter plot with density contours showed a saddle shape with two distinct peaks, hereby referred to as “Child C1” (n = 27) and “Child C2” (n = 23), respectively. C1 appeared on the upper left of the chart, occupying a more dispersed space, while C2 appeared on the lower right, with a more compact pattern (Fig. 3D).

The loading scores (eigenvectors on which the data projected; Methods) for the first two PCs showed different projecting weights and directions for the 57 items surveyed (Fig. 3E). In particular, on the first PC, various items measuring the physical capabilities were the most positively weighted domain, while psychological metrics dominated the other, negative, end (Fig. 3E; left). This translated to higher scores of mobility for the patients projected to the C2 cluster, and higher scores for psychological distress towards the negative end of PC1 (Fig. 3D). The loading scores for PC2 were less stratified, with a stronger presence of physical metrics (mobility) towards the negative end (Fig. 3E; right). The loading scores of PC1 and PC2 suggested that C1 patients suffered from higher disease burden and worse functional mobility.

Using hierarchical clustering, we scored each item with dendrograms showing the sample-sample and item-item correlations (Fig. 3F). The horizontal axis of the heatmap, consisting of 57 PROMIS items, showed that similar categories were clustered together. We noted that the patients were categorized in a similar topological pattern as shown in the PCA plot. The marginal distributions showed more ‘floor’ and ‘ceiling’ effects (bimodal distributions) from the C2 patients, reflecting their optimism in their physical and psychological states (Fig. 3F). The C1 patients tended to have unimodal evaluations, suggesting their overall pessimism in self-evaluations (Fig. 3F).

### Parental PROMIS reflected similar psychophysical stratification of children

**Fig. 4:**
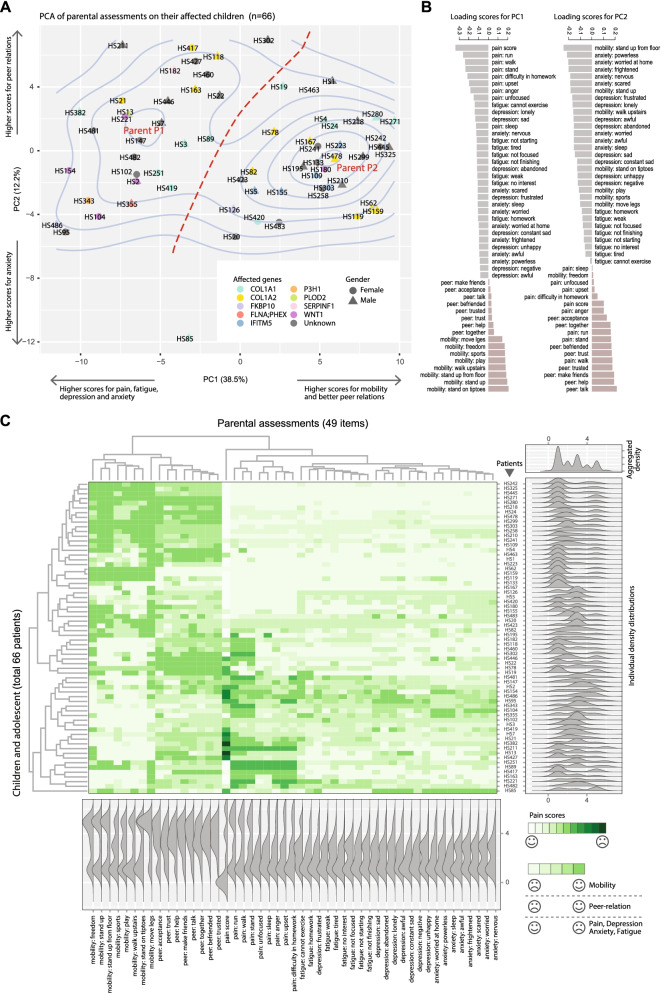
PCA and clustering analyses of parental PROMIS data. **A** Scatter plot showing the first two PCs, with density contours. Red curves indicate saddle and valley between the two peaks/clusters. **B** Bar charts showing the ordered loading scores for the first and second PC. **C** Heatmap with clustering showing data in their original values. Marginal ridge plots show the marginal densities either per-patient (row-wise) or per PROMIS-item (column-wise), with an aggregated density for all data points placed on the top right corner. The meanings of the colors were explained by the smile or sad face symbols. The PROMIS items are abbreviated by their categories and a representative keyword. Refer to Additional file 1 for corresponding questions in full

**Fig. 5:**
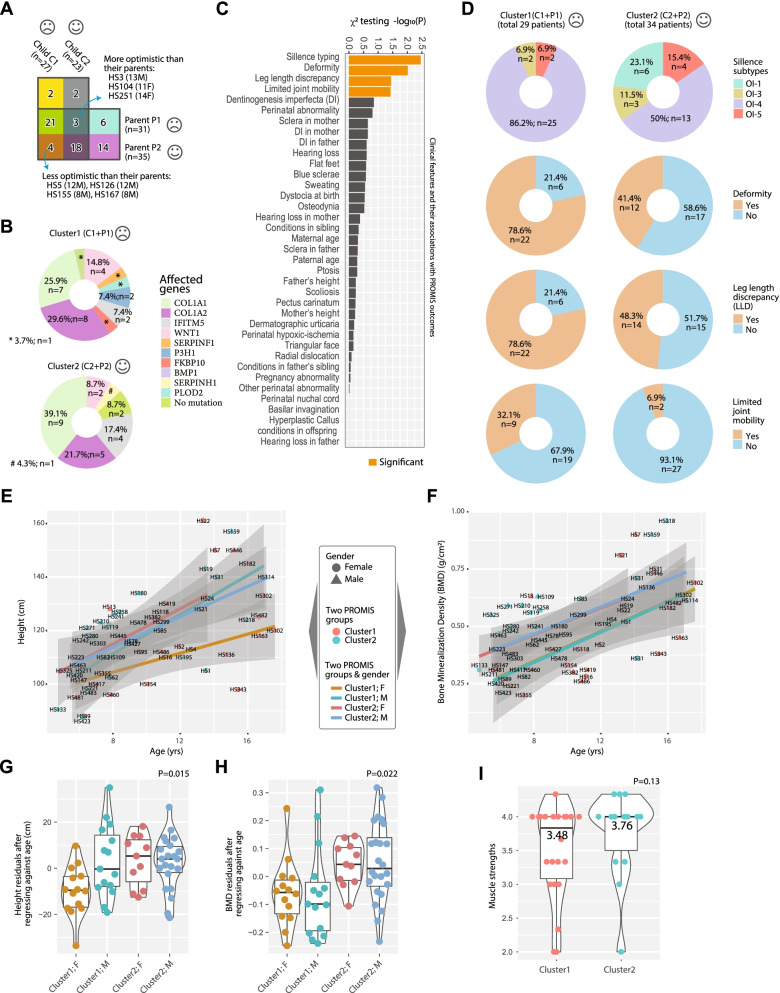
The identification of two PROMIS groups in child and adolescent patients, and clinical characteristics of the groups. **A** A Venn diagram showing the identification of two PROMIS groups in the young patients by their own assessments and their parents’ assessments. The numbers correspond to the numbers of patients in each category. The labels C1, C2, P1 and P2 correspond to the Figs. 3D and 4A. The 7 patients with discordance between the patients’ own and their parents’ assessments were considered “ambiguous”. **B** Pie charts showing the distribution of affected genes in the two groups of patients. **C** Bar-charts showing the clinical features in decreasing order of significance, in terms of their associations with the PROMIS groups. **D** Pie charts showing the positive rates for each of the four significantly associated clinical features in the two PROMIS groups. **E** Scatter plot showing the patients’ heights versus their ages. The straight lines are regression curve fitted for each of the four patient groups. **F** Scatter plot showing the patients’ spine BMD versus their ages. The straight lines are regression curve fitted for each of the four patient groups. **G** Violin plots showing the residuals of heights after fitting against age, with respect to the four groups. **H** Violin plots showing the residuals of BMDs after fitting against age, with respect to the four groups. *P* values in **G**, **H** indicate the F-testing result. **I** Violin plot showing muscle strengths in the two clusters

To evaluate the validity of PROMIS stratification from the children and adolescents, we also conducted questionnaire surveys on their parents. In total, 49 items were evaluated from the parents of 66 patients, and similar analyses were conducted as above. Remarkably, two clusters were also identified, and are hereby referred to as P1 and P2, which included 31 and 33 parents, respectively (Fig. 4A). The P2 cluster was more compact with denser contours. The top two PCs captured over half of the data variance. PC1, the major component that distinguished the two populations, was positively correlated with mobility and peer relations, but negatively correlated with pain, depression and other psychological distress (Fig. 4B). As such, P1 was considered as an “unhappy/pessimistic” group, and P2 as a “happy/optimistic” group. Hierarchical clustering simultaneously recapitulated the sample-sample relations in the PCA plot (Fig. 4C). The marginal distributions in the parental heatmap also displayed highly similar patterns as in the self-assessments of the young patients, with P2 patients showing stronger unimodal distributions, and P1 otherwise (Fig. 4C). To delineate the consistency between the parental and self-assessed outcomes, we presented a Venn diagram, showing 84.8% (39 out of 46) concordance between these two datasets (Fig. 5A). The remarkable consistency in terms of the overall patterns of clustering between parental and children’s self-assessments further supported the validity of the PROMIS instruments.

### Two clusters of patients present with different genetic and phenotypic features

We asked if the subjective evaluations and classification are related to objective factors such as genetics, clinical status and socioeconomic status. We grouped all the unambiguous cases into Cluster 1 (29 patients) and Cluster 2 (34 patients), and referred to them as the “unhappy/pessimistic” and “happy/optimistic” clusters, respectively (Fig. 5A). Surprisingly, no statistical association was detected between genotypes and the two clusters (χ^2^ *P* = 0.36), which may be due to the fact that the mutational spectra are so diverse (Additional file 3) that information of the affected genes alone is not sufficient to predict patient outcomes, especially given the relatively small sample size here (n = 63). We noted that there were three times more patients carrying recessive mutations, including in *WNT1*, *FKBP10*, *P3H1, BMP1,* and *SERPINF1*, in Cluster 1 (n = 9) than in Cluster 2 (n = 3) (Fig. 5B), which is consistent with the more severe skeletal phenotypes in autosomal recessive OI [37]. Secondary clinical features, including blue sclera, hearing loss and dentinogenesis imperfecta, are usually present in OI patients [1]. By Pearson’s ${\chi }^{2}$ association test, we found that four items were distributed with statistical significance, including clinical subtyping (Sillence scale), deformity, leg length discrepancy (LLD) and limited joint mobility (Fig. 5C). In particular, type IV OI was strongly enriched (86.2% in Cluster 1 vs. 50.0% in Cluster 2, χ^2^ *P* = 0.004), as was type I (23.1% in Cluster 2 vs. 0% in Cluster 1, χ^2^ *P* = 0.013). Type I OI is the mildest form of the disease and Type IV ranges from moderate to progressive deforming [1, 10, 38]. Deformity and LLD showed a similar trend, with positive rates in Cluster 2 almost double those in Cluster 1. The positive rate of joint abnormality was increased by 4.6 times, from 6.9% in Cluster 2 to 32.1% in Cluster 1 (Fig. 5D), limiting the physical capacity of patients and causing major difficulties in daily life.

We further investigated the correlations between the clustering and other documented medical quantities including height and bone mineral density (BMD). Linear regression analysis was performed between the height and age in a gender- and cluster-specific manner. We found that the fitted curve for the females in Cluster 1 vastly deviated from that of the other three groups, while the BMD of both genders was consistently lower in Cluster 1 than in Cluster 2 (Fig. 5E, F). To reduce the influence of age, we performed regression analysis of heights and BMD against ages before statistical analyses (Methods). The result showed a significant association between age-corrected heights and gender/clusters (*P* = 0.015, ANOVA). Post hoc analyses by Tukey’s honestly significant difference test showed the biggest difference between Cluster1F and Cluster2M (*P* = 0.028), followed by Cluster1F-vs-Cluster1M (*P* = 0.032) and Cluster1F-vs-Cluster2F (*P* = 0.052). Comparison of age adjusted BMD between the two clusters indicated that both genders showed lower bone density in Cluster 1 as compared with Cluster 2 (*P* = 0.022, ANOVA) (Fig. 5H). Tukey’s honestly significant difference test showed that the adjusted *p*-value between Clusters 1 and 2 was 0.002, and between genders was 0.54. No statistical difference was found between the two clusters in terms of muscle strengths (*P* = 0.13), although trend-wise Cluster 2 (average strength 3.76) did have better scores than Cluster 1 (average strength 3.48) (Fig. 5I). In brief, the physical characteristics of the patients, including limb deformity, height and BMD, were the most relevant predictors for the two-cluster classification as revealed by the PROMIS data.

### Longitudinal data confirms consistency of two-cluster stratification

**Fig. 6:**
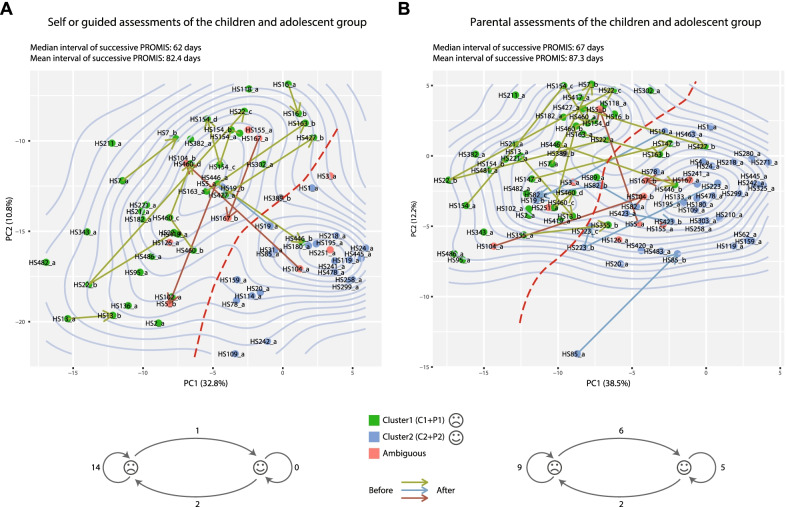
Projections of later PROMIS outcomes to the PCA of the first PROMIS outcome. **A** Projections of children and adolescent’s later PROMIS outcomes (second, third or fourth) on the loading scores of the PCA of the first PROMIS outcomes. **B** Projections of parents’ later PROMIS outcomes (second, third or fourth) on the loading scores of the PCA of the first PROMIS outcomes. Arrows point from the PROMIS outcome at one time-point to the next, for each patient. Arrow colors indicate the original states in the first PROMIS. The transition diagrams at the bottoms of **A**, **B** show the numbers of state changes for each pair of successive PROMIS surveys. The numbers on the edges show the numbers of patients or numbers of times a patient changes from one state to another (or the same) state

Both the young patients and their parents were invited to join our longitudinal surveys, with intervals averaging about three months (82.4 and 87.3 days for the children and their parents, respectively) (Additional file 2). We projected these PROMIS data into the loading scores for the top principal components of the first PROMIS dataset (Methods). For the self-assessed data, we observed a much greater frequency of Cluster 1 patients involved in the longitudinal study, as indicated by the green arrows (Fig. 6A). This was consistent with the overall poorer conditions in this population, which may require more frequent hospitalizations. We also observed an overwhelming propensity for these patients to stay in the same unhappy state even after clinical interventions (14 out of 15 patients) (Fig. 6A). A similar but slightly encouraging trend can be observed from the parental longitudinal data, where the numbers of state-changes from bad to good (n = 6) was 3 times more than the other way round (n = 2) (Fig. 6B). These longitudinal data confirmed the consistency of diversity among young OI patients’ subjective evaluation, providing treatment decision and disease management recommendations for the clinicians and caregivers.

### A spectrum of physical capabilities among adult patients

**Fig. 7:**
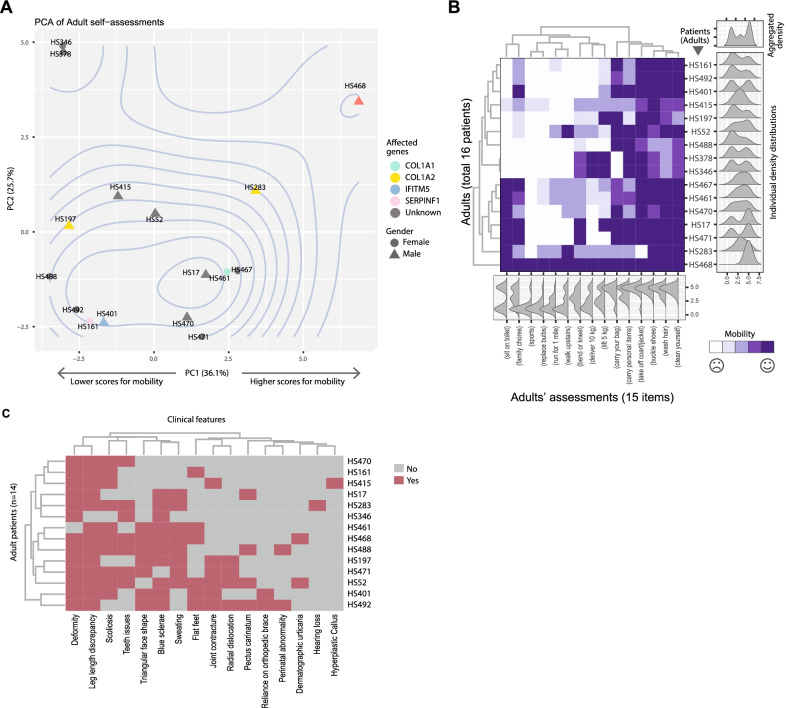
PROMIS outcomes of adult patients. **A** PCA of adult patients’ PROMIS outcomes. **B** Heatmap showing the individual assessment outcomes. Ridge plots to the right and bottom are the density estimations that show the row-wise or column-wise distributions. **C** Heatmap showing the clinical features of the adult patients. The PROMIS items are abbreviated by their categories and a representative keyword. Refer to Additional file 1 for corresponding questions in full

In addition to child and adolescent data, we conducted a relatively small-scale survey on 16 adult patients regarding their physical capabilities only. PCA analyses showed that the adult patients were enriched in one major cluster only (Fig. 7A). Heatmap analyses of the original scores and phenotypic features showed continuous spectra among these patients, without clear clustering patterns (Fig. 7B, C).

## Discussion

Traditionally, medical services and healthcare primarily focused on genetics, diagnostics, pharmacologic treatment, and orthopedic surgeries, all defined by objective metrics, to address the deformity and fracture issues of OI patients. It is gradually recognized that for a lifelong condition like OI, what ultimately matters most is patients’ quality of life (QoL) from their own perspective (the ‘subjective’ criteria).

The clinical manifestations of OI represent a continuum ranging from mild or moderate, to severe or perinatal lethal, which is in line with the diverse mutational spectrum observed in this condition [35]. Genetic mutations affecting collagen production, conformation and osteoblast differentiation cause skeletal deformity and joint dysfunction, leading to different levels of functional limitation and physical disability [39]. Pain/discomfort and functional mobility have been reported to be the most problematic domains for patients with OI, fibrous dysplasia and other skeletal disorders [18]. Knowledge of how chronic diseases affect health-related QoL may provide the grounds for improving treatments and support to the needs of these patients. Several studies have leveraged different generic tools to assess health related QoL in children and adults with OI [16, 18, 22]. The measures across these studies may differ, thus preventing direct comparisons and meta-analyses. On the other hand, using generic tools may not provide information on OI-specific aspects affecting patients’ QoL, such as pain and social relations [40]. Therefore, it is highly recommended that comprehensive assessment of QoL should include perspectives from both the children and their parents as the ground reference for clinical practice and research.

In this study, we probed into the subjective quantitation of patient reported outcomes, identified two major clusters of patients with vastly distinct outlooks on life, one (C2) being more optimistic than the other (C1). In addition, we established a connection between clinical phenotypes and patient psychological status. We found clinical subtyping, bone deformity, leg length discrepancy and limited joint mobility were most relevant to the patients’ PROMIS assessments. Specifically, patients with such issues were much more enriched in the “unhappy” cluster (C1), which coupled with QoL meta-analyses suggesting association of pain, scoliosis and participation restriction with low QoL in OI [22]. The reliability of this dichotomized clustering was further supported by two additional sources of information: first, the simultaneous survey outcomes of the children and their parents; second, longitudinal data. In the former, a minimum concordance of 84.8% (39 out of 46) was estimated between parent and child surveys. In the longitudinal data, we found that the”unhappy” individuals tend to stay “unhappy” after multiple surveys, which unfortunately seems to suggest a grim situation whereby treatments often fail to improve QoL. It is also possible that the follow-up intervals (~ 3 months) were short or insufficient to show the benefits of treatment. Thus, multiple and longer intervals may be needed in future longitudinal studies. We noted some of the question items were similar to each other, which has been well addressed by applying de-correlation methods (PCA) in this study.

The widely-used PROMIS instrument was validated by a pilot study [25]. By leveraging the PROMIS instruments, we presented more systematic, in-depth analyses by overcoming some of their limitations, including gender disparity, insufficient ethnic diversity and lack of self-assessments in the children [25]. We conducted both parental and self-assessments, observing a high consistency between them. Longitudinal data further confirmed the clustering analyses, which also helped to evaluate the changes of life quality over time in OI individuals. We performed genetic testing on 72 of the 90 patients, and detected pathogenic variants in 11 OI-related genes. But we detected no significant association between genotypes and PROM-based stratification, which may be due to the wide spectrum of mutations in OI. The future direction should focus on the development/expansion of disease specific measures in the PROMIS database to detect individual health status and life quality of OI patients as a basis for evaluating clinical management and health maintenance.

Notwithstanding, we are aware that multiple aspects can be improved. The cohort was not randomized. The patients were surveyed consecutively on a first come, first served basis. As with most other QoL/PROMIS studies, no control group was available. A control group of non-diseased people or of other diseases would be beneficial to compare individual PROMIS items to a baseline background; although it can be projected such a control group would tend to display “floor” and “ceiling” effects for most items. A larger cohort is envisioned to enhance statistical power, especially in light of the multiple clinical features being tested against the stratification results, although sample size is always a big challenge with rare diseases such as OI. The psychological wellbeing in the adult patients was not comprehensively examined in our study. Advisable next-steps include supplementing recruitment from the OI community, multi-center cooperation, standardized clinical categorization of disease severity and an OI-specific questionnaire.

## Conclusion

In this study, we obtained PROM data regarding the disease experiences from young OI patients and their parents, as well as their longitudinal follow-ups to assess the physical and psychological health status and responses to clinical interventions. With an advanced analytical framework, we stratified patients into two major groups, which we showed were associated with clinical manifestations, including leg length discrepancy and limited joint mobility. More severe phenotypes tended to associate with greater psychological pessimism. A high consistency of stratification patterns between the affected children and their parents further validated the PROM stratification. The longitudinal data also showed that patients tended to stay in the same psycho-physical states. The lack of genotype-PROM association may be due to the relatively small sample size in this study and the diverse mutation spectrum in OI. Our study demonstrates the merits of in-depth analyses in patient reported outcomes to understand the diverse clinical phenotypes and psycho-physical health in OI patients.

## Supplementary Information

**Additional file 1.** PROMIS forms used in this study for child patients and their parents, and for adult patients.

**Additional file 2.** Additional demographical characteristics of the current OI cohort.

**Additional file 3.** The mutation spectrum of OI related genes in 72 patients of the cohort.

## Data Availability

Some of the custom scripts for data processing were deposited at: https://github.com/HKUSZH/OI-PROMIS. Please contact authors for data requests.
