# Supplementary material for: Patient-reported outcomes in a Chinese cohort of osteogenesis imperfecta unveil psycho-physical stratifications associated with clinical manifestations
Source: Orphanet J Rare Dis. 2022 Jun 28;17:249. doi: 10.1186/s13023-022-02394-7 (PMC9238011; doi:10.1186/s13023-022-02394-7)
Supplement: Supplementary file 2 — Additional file 2. Additional demographical characteristics of the current OI cohort. [file 13023_2022_2394_MOESM2_ESM.pdf]

Additional file 2

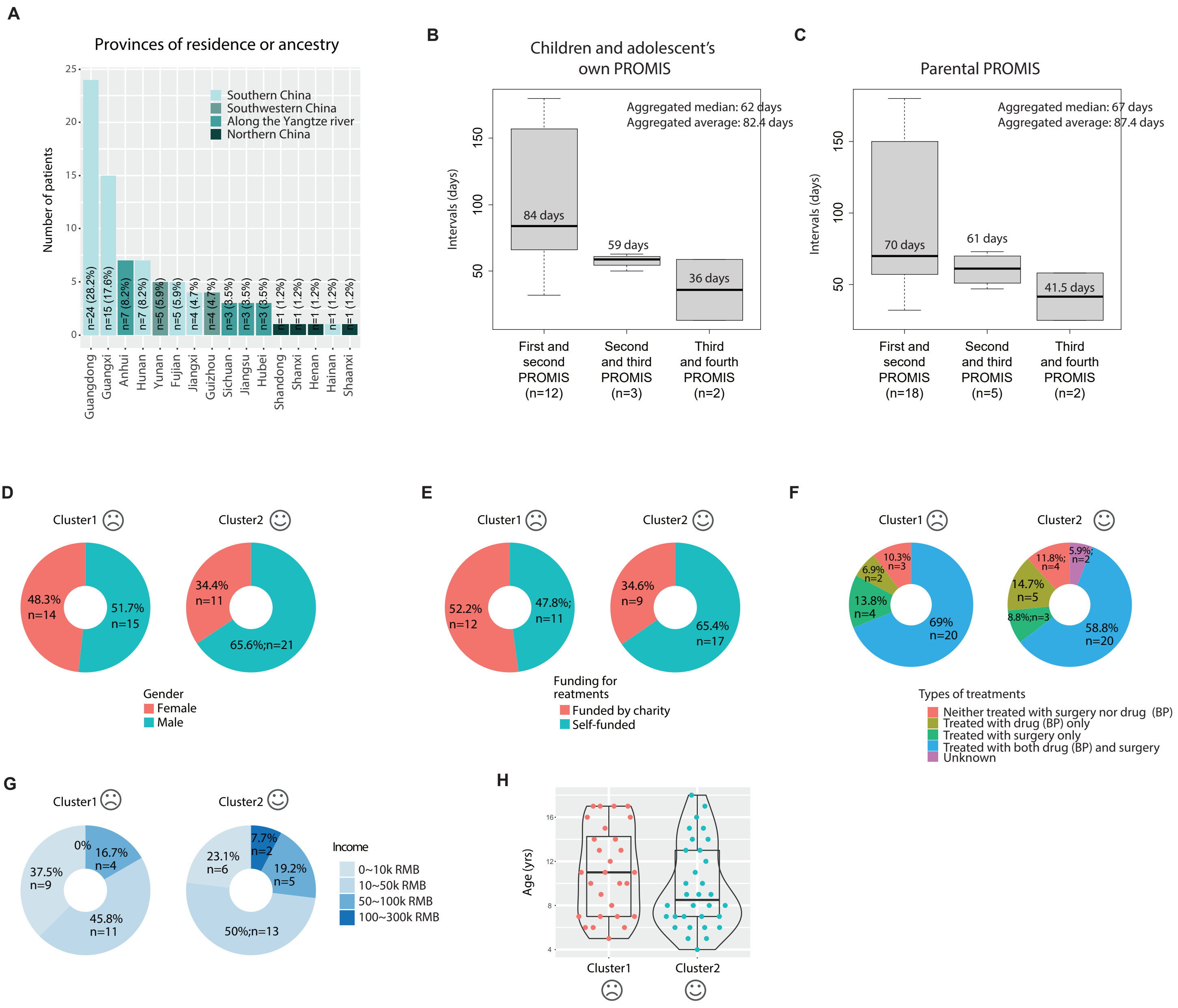

Additional file 2. Additional demographical characteristics of the current OI cohort.
A, Provincial/regional distribution of the current OI cohort.
B, Intervals of successive PROMIS survey in the longitudinal data of the children.
C, Intervals of successive PROMIS survey in the longitudinal data of the parents.
D, Gender distribution in the two clusters of patients identified.
E, Sources of funding for treatment in the two clusters of patients identified.
F, Genotypic distributions for treatment in the two clusters of patients identified.
G, Family income in the two clusters of patients identified.
H, Age distributions in the two clusters of patients identified.
