# Supplementary material for: Patient-reported outcomes in a Chinese cohort of osteogenesis imperfecta unveil psycho-physical stratifications associated with clinical manifestations
Source: Orphanet J Rare Dis. 2022 Jun 28;17:249. doi: 10.1186/s13023-022-02394-7 (PMC9238011; doi:10.1186/s13023-022-02394-7)
Supplement: Supplementary file 3 — Additional file 3. The mutation spectrum of OI related genes in 72 patients of the cohort. [file 13023_2022_2394_MOESM3_ESM.pdf]

Additional file 3. The mutation spectrum of OI related genes in 72 patients of the cohort.

IDs	Sillence Type	Affected Genes	Novel	Nucleotide change	Predicted amino acid change	Inheritance	Mutation effect
HS89*	4	COL1A1	No	c.2461G>A	p.Gly821Ser	AD†	missense
HS460*	4	COL1A1	No	c.2461G>A	p.Gly821Ser	AD	missense
HS420*	4	COL1A1	No	c.2461G>A	p.Gly821Ser	AD	missense
HS419*	4	COL1A1	No	c.2461G>A	p.Gly821Ser	AD	missense
HS103*	4	COL1A1	No	c.3123delC	p.Ala1042Leufs*66	AD	frameshift
HS251*	1	COL1A1	YES	c.3870C>A	p.Phe1290Leu	AD	missense
HS271*	1	COL1A1	YES	c.1298_1299insT	p.Gly434Argfs*2	AD	frameshift
HS280*	4	COL1A1	No	c.757C>T	p.Arg253*	AD	nonsense
HS382*	4	COL1A1	No	c.2398-1G>C	Splicing	AD	splicing
HS373*	1	COL1A1	No	c.1001dupC	p.Gly335Trpfs*18	AD	frameshift
HS85*	4	COL1A1	No	c.3207+1G>A	splicing	AD	splicing
HS3*	4	COL1A1	No	c.1669-2A>G	splicing	AD	splicing
HS355*	4	COL1A1	YES	c.1145C>A	p.Ala382Asp	AD	missense
HS427*	4	COL1A1	YES	c.3468C>G	p.Asn1156Lys	AD	missense
HS470*	3	COL1A1	No	c.2155G>A	p.Gly719Ser	AD	missense
HS488*	3	COL1A1	No	c.2155G>A	p.Gly719Ser	AD	missense
HS22*	4	COL1A1	No	c.1252delT	p.Ser418Leufs*123	AD	frameshift
HS492*	4	COL1A1	No	c.934C>T	p.Arg312Cys	AD	missense
HS468	1	COL1A1	No	c.3207+1G>A	splicing	AD	splicing
HS119	1	COL1A1	No	c.1866delT	p.Gly623Alafs*143	AD	frameshift
HS241	1	COL1A1	No	c.432delC	p.Gly145Aspfs*120	AD	frameshift
HS242	1	COL1A1	No	c.432delC	p.Gly145Aspfs*120	AD	frameshift
HS4	4	COL1A1	Yes	c.3351G>T	p.Gln1117His	AD	missense
HS31	1	COL1A1	No	c.769G>A	p.Gly257Arg	AD	missense
HS482*	4	COL1A2	No	c.792+1insT	splicing	AD	splicing
HS481*	1	COL1A2	No	c.792+1insT	splicing	AD	splicing
HS167*	4	COL1A2	No	c.838G>C	p.Gly280Arg	AD	missense
HS163*	4	COL1A2	No	c.838G>C	p.Gly280Arg	AD	missense
HS78*	4	COL1A2	No	c.792+1G>A	splicing	AD	splicing

HS21*	4	COL1A2	No	c.1801G>A	p.Gly601Ser	AD	missense
HS389*	3	COL1A2	YES	c.2197G>A	p.Gly733Ser	AD	missense
HS62*	1	COL1A2	No	c.1927G>A	p.Gly643Arg	AD	missense
HS82*	4	COL1A2	No	c.3304G>T	p.Gly1102Cys	AD	missense
HS159*	4	COL1A2	No	c.1801G>A	p.Gly601Ser	AD	missense
HS118*	4	COL1A2	No	c.2441G>A, c.4048G>A	p.Gly814Glu/p.Gly1350Ser	AD	missense
HS417*	4	COL1A2	No	c.1991G>A	p.Gly664Asp	AD	missense
HS197*	4	COL1A2	No	c.2035G>A	p.Gly679Ser	AD	missense
HS16*	4	COL1A2	YES	c.1990G>T	p.Gly664Cys	AD	missense
HS302*	4	COL1A2	YES	c.3237_3238insCGAGGCCCTCAGGGTCAC	p.Ile1079delinsIRGPQGH	AD	in-frame insertion
HS346*	4	COL1A2	No	c.693+5G>A	splicing	AD	splicing
HS17	4	COL1A2	YES	c.1990G>T	p.Gly664Cys	AD	missense
HS283	4	COL1A2	No	c.1801G>A	p.Gly601Ala	AD	missense
HS478	1	COL1A2	No	c. 3197G>T	p. Gly1066Val	AD	missense
HS155	5	IFITM5	No	c.-14C>T	p.Met1ext-5	AD	new start codon
HS401	5	IFITM5	No	c.-14C>T	p.Met1ext-5	AD	new start codon
HS136	5	IFITM5	No	c.-14C>T	p.Met1ext-5	AD	new start codon
HS223	5	IFITM5	No	c.-14C>T	p.Metlext-5	AD	new start codon
HS5	5	IFITM5	No	c.-14C>T	p.Met1ext-5	AD	new start codon
HS303	5	IFITM5	No	c.-14C>T	p.Met1ext-5	AD	new start codon
HS463	5	IFITM5	No	c.-14C>T	p.Met1ext-5	AD	new start codon
HS20	5	IFITM5	No	c.-14C>T	p.Met1ext-5	AD	new start codon
HS95	5	IFITM5	No	c.-14C>T	p.Met1ext-5	AD	new start codon
HS104	4	WNT1	No	c.216dupA, c.571G>A	p.Arg73Thrfs*82, p.Gly191Arg	AR	frameshift/missense
HS147	4	WNT1	No	c.620G>A	p.Arg207His	AR	missense
HS2	4	WNT1	No	c.557A>T	p.Asp186Val	AR	missense
HS221	4	WNT1	No	c.677C>T	p.Ser226Leu	AR	missense
HS133	4	WNT1	No	c.104+1G>A, c.501G>C	splicing, p.Trp167Cys	AR	splicing/missense
HS180	4	WNT1	No/Yes	c.677C>T, c.877G>A	p.Ser226Leu, p. Glu293Lys	AR	missense
HS154	4	WNT1	No	c.466delC	p.R156Gfs*42	AR	frameshift
HS182	4	SERPINF1	No	c.72dupC	p.Glu27Glyfs*38	AR	frameshift

HS161	3	SERPINF1	No	c.259_260insCGGCCCTCT	insertion	AR	in-frame insertion
HS423	4	SERPINH1	No	c.56_57insCGAGGTGAAGAAACCTGCAG, c.461A>G	p.Ala27Argfs*2, p.Tyr154Cys	AR	missense/framshift
HS486	4	FKBP10	No	c.918-3C>G	splicing	AR	splicing
HS471	3	FKBP10	No	c.745C>T, c.825dupC	p.Gln249*, p.Gly278Argfs*95	AR	nonsense/frameshift
HS343	3	P3H1	No	c.2164C>T	p.Gln722*	AR	nonsense
HS211	4	P3H1	No/No	c.105_120delTGATCTGCTCTTCGCC, c.2164C>T	p.Asp36Argfs*16, p.Gln722*	AR	frameshift/nonsense
HS415	3	BMP1	No	c.2183dupC	p.Gln730Profs(no Ter)	AR	frameshift
HS7	4	BMP1	No	c.625G>A, c.1814C>T	p.Gly209Ser, p.Pro605Leu	AR	missense
HS13	4	PLOD2	No	c.2051A>T, c.1975C>T	p.Asp684Val, p.Arg659*	AR	missense/nonsense
HS52	1	No mutation	No	--	--	--	--
HS19	4	No mutation	No	--	--	--	--
HS325	4	No mutation	No	--	--	--	--

* Published in our recent work [1].

† AD: autosomal dominant. AR: autosomal recessive.

1. Chen, P., et al., *Phenotypic Spectrum and Molecular Basis in a Chinese Cohort of Osteogenesis Imperfecta With Mutations in Type I Collagen.* Front Genet, 2022. **13**: p. 816078.
